# Community-Level Social Support to Mitigate the Impact of Combined Frailty and Multimorbidity on Psychological Distress Among Rural Chinese Older Adults During the COVID-19 Pandemic: Multilevel Modeling Study

**DOI:** 10.2196/43762

**Published:** 2023-03-09

**Authors:** Yi Wang, Peipei Fu, Jie Li, Tingting Gao, Zhengyue Jing, Qiong Wang, Dan Zhao, Chengchao Zhou

**Affiliations:** 1 Centre for Health Management and Policy Research School of Public Health Cheeloo College of Medicine, Shandong University Jinan China; 2 National Health Commission Key Lab of Health Economics and Policy Research Shandong University Jinan China

**Keywords:** psychological distress, frailty, multimorbidity, community-level social support, COVID-19 pandemic, psychological, rural, older adults, community, support, effectiveness

## Abstract

**Background:**

Accumulating research provides evidence that the psychological health of older people deteriorated from before to during the COVID-19 pandemic. Unlike robust individuals, coexisting frailty and multimorbidity expose older adults to more complicated and wide-ranging stressors. Community-level social support (CSS) is also an important impetus for age-friendly interventions, and it is 1 of the components of social capital that is seen as an ecological-level property. To date, we have not found research that examines whether CSS buffered the adverse impacts of combined frailty and multimorbidity on psychological distress in a rural setting during COVID-19 in China.

**Objective:**

This study explores the combined effect of frailty and multimorbidity on psychological distress in rural Chinese older adults during the COVID-19 pandemic and examines whether CSS would buffer the aforementioned association.

**Methods:**

Data used in this study were extracted from 2 waves of the Shandong Rural Elderly Health Cohort (SREHC), and the final analytic sample included 2785 respondents who participated in both baseline and follow-up surveys. Multilevel linear mixed effects models were used to quantify the strength of the longitudinal association between frailty and multimorbidity combinations and psychological distress using 2 waves of data for each participant, and then, cross-level interactions between CSS and combined frailty and multimorbidity were included to test whether CSS would buffer the adverse impact of coexisting frailty and multimorbidity on psychological distress.

**Results:**

Frail older adults with multimorbidity reported the most psychological distress compared to individuals with only 1 or none of the conditions (β=.68, 95% CI 0.60-0.77, *P*<.001), and baseline coexisting frailty and multimorbidity predicted the most psychological distress during the COVID-19 pandemic (β=.32, 95% CI 0.22-0.43, *P*<.001). Further, CSS moderated the aforementioned association (β=–.16, 95% CI –0.23 to –0.09, *P*<.001), and increased CSS buffered the adverse effect of coexisting frailty and multimorbidity on psychological distress during the COVID-19 pandemic (β=–.11, 95% CI –0.22 to –0.01, *P*=.035).

**Conclusions:**

Our findings suggest that more public health and clinical attention should be paid to psychological distress among multimorbid older adults with frailty when facing public health emergencies. This research also suggests that community-level interventions prioritizing social support mechanisms, specifically improving the average levels of social support within communities, may be an effective approach to alleviate psychological distress for rural older adults who concurrently manifest frailty and multimorbidity.

## Introduction

Mental health is an important component of healthy aging, and reducing psychological distress is vital for both physical health and well-being in later life [1]. Accumulating research [2-4] provides evidence that the psychological health of older people deteriorated from before to during the COVID-19 pandemic. It is important to identify groups that are at greater risk of psychological distress in response to the COVID-19 pandemic so as to develop appropriate interventions, especially for those who live in rural areas, because rural older persons have lower socioeconomic status, have less access to social support and health services, and report worse mental health compared with those in urban areas [5]. Frailty and multimorbidity are common and major age-related clinical syndromes and well-established independent risk factors for psychological health in older people [6-8]. Separate studies have reported that frailty or multimorbidity can independently increase the levels of psychological distress in older people [7,9,10]. However, an individual can be multimorbid but not frail or frail while having multimorbidity. More importantly, frailty can be more modifiable than chronic conditions [11], and this distinction is important when assessing relationships with psychological health because they may inform different management strategies for prognosis and planning interventions. Both the National Institute for Health and Care Excellence (NICE) and the British Geriatrics Society emphasize the importance of combined frailty and multimorbidity in older adults who are at greater risk of adverse outcomes and might benefit more from treatment optimization [12,13]. For example, according to the statement document of NICE, not all older adults with multimorbidity require additional support beyond standard health care but frailty should be considered an important condition to keep in mind in the management of older adults with multimorbidity [13,14]. Unlike robust individuals, coexisting frailty and multimorbidity would expose older adults to more complicated and wide-ranging stressors. Nevertheless, the combined effect of frailty and multimorbidity on psychological distress has not been previously investigated in community-dwelling older adults in rural China. By analyzing whether combined multimorbidity and frailty contribute to psychological distress can inform the development of interventions for those at heightened risk and aid policy decisions concerning the subsequent management of the COVID-19 pandemic response.

The social determinants of the health framework [15] believe that health inequality cannot be attributed solely to differences in individual characteristics. The environmental contexts that reflect the social resources and opportunities can affect individuals’ access to healthy lives and choices strongly and ultimately shape our health and well-being [16]. Understanding the contextual determinants of psychological distress has important public health implications for developing population-scale interventions. Just as biologists have distinguished levels of organization to describe living organisms, social epidemiologists have advanced levels to delineate different scales of environmental contexts that affect an individual’s health, such as the community level or state level [17,18]. Among these different levels of contexts, the community is 1 of the most common areas of focus in China. The concept of “community” was adopted by the Chinese government through the community-building project since the 1980s, which can be understood as a social group comprising people living in the same area, sharing similar values or culture, and having direct or indirect continuous interaction. Communities in rural China exert important functionalities in providing primary health care services, advocating government policies, managing social life affairs, and improving quality and cultural accomplishment for community residents [19]. In China’s response to COVID-19, the Chinese government implemented social distancing and lockdown measures at the community level, which has become the intersection and bridge between government institutions, businesses, social organizations, volunteer groups, families, and individuals [20]. The psychological health of older people living in rural areas may be particularly sensitive to community social environments as they tend to be less mobile and rely more on locally provided services, as well as social support and connection. Community-level social support (CSS), defined as the extent to which a community leads someone to be cared for, loved, respected, and be a member of a network of mutual obligations [21], has been associated prominently with overall health and longevity [22,23]. Thus, CSS may serve as a potentially important target of intervention on psychological health.

Social capital theory [24] provides a useful framework for understanding the potentially protective mechanisms of increased CSS on psychological distress theoretically. CSS, as 1 of the components of social capital that is seen as an ecological-level property, can give rise to social capital that reflects the levels of reciprocity and connectedness within a community [24], and thus exerts a contextual effect on individual health via several possible pathways, such as dissemination of health-promoting knowledge, maintenance of healthy norms of behavior through informal social control, promotion of access to local services and amenities, and provision of psychological processes of emotional support and mutual respect [25,26]. Although individual-level studies of social support have yielded useful insights [27-29], an examination of social support as a contextual effect on reducing psychological distress remains understudied. Previous studies have highlighted the protective effects of CSS on the mental health and well-being of older people [30]; however, no such research has been conducted in rural China. People living in rural areas usually have more difficulties in accessing public services and lack of medical attention compared with those in urban areas [31] and thus may experience more psychological distress. Further, CSS may not only affect mental health directly but also have indirect effects by serving as a buffer against the adverse impacts of different stressors, such as frailty and multimorbidity. To date, we have not found research that examines whether CSS buffered the adverse effects of combined frailty and multimorbidity on psychological distress in a rural setting during the COVID-19 in China. A better understanding of the cross-level interaction between CSS and combined frailty and multimorbidity is necessary to design more appropriate and precise interventions aimed at reducing psychological distress.

Taken together, the first aim of our study is to explore the longitudinal association between coexisting frailty and multimorbidity and psychological distress in rural older adults, as well as whether baseline combined frailty and multimorbidity predict psychological distress during the COVID-19 pandemic. The second aim of this study is to examine whether improving CSS would buffer the adverse impacts of coexisting frailty and multimorbidity on psychological distress.

## Methods

### Study Design and Data Sources

Data used in this study were extracted from 2 waves of the Shandong Rural Elderly Health Cohort (SREHC), an ongoing longitudinal study of community-dwelling older people in rural Shandong, China. The baseline survey was completed in June 2019, which was considered prior to the COVID-19 pandemic on the basis of the declaration from the World Health Organization on March 11, 2020, that COVID-19 can be characterized as a pandemic [32]. The detailed study design, sampling, and data collection have been described elsewhere [2,33,34]. Briefly, we used the multistage stratified random sampling method to select participants, excluding those who had a clinical diagnosis of dementia or psychiatric diseases, could not complete the interview due to severe physical conditions, or were unwilling to cooperate with the interviewers. There were 3243 respondents aged 60 years and above who participated in the baseline survey. Next, a follow-up survey was conducted during the COVID-19 pandemic from August to September 2020. The final analytic sample included 2785 (85.88%) respondents who participated in both baseline and follow-up surveys. We restricted the analysis to these 2 data points for the following reasons: First, we were specifically interested in examining the short-term consequences of combined frailty and multimorbidity on psychological distress so as to identify the most vulnerable group with the greatest risk of worse psychological outcomes and inform early interventions during the pandemic. Second, previous research has shown that social support would have an immediate effect on a scaled measure of psychological health [27]; therefore, we assessed the moderating role of CSS between combined frailty and multimorbidity and psychological distress with just a 1-year lag based on the assumption that there may be a limited time lag for CSS to mitigate one’s psychological distress.

### Ethical Considerations

The study purpose, significance, methods, and risks were explained to all participants, and written informed consent was obtained from each participant before the 2 surveys. Ethical approval for the study was obtained from the Ethics Committee of Shandong University (approval no. 20181228).

### Measurements

We used the 10-item Kessler Psychological Distress Scale (K10) [35] to assess the psychological distress in this study. K10 is a commonly global measure for screening mental health, and its reliability and validity have been confirmed among older people in China [36,37]. K10 contains 10 items using a 5-point Likert scale from “none of the time” to “all of the time” to evaluate psychological distress, including depression, anxiety, nervousness, hopelessness, restlessness, and worthlessness, in the past 4 weeks. K10 in this study showed good internal consistency reliability, with Cronbach α=.91 for both baseline and follow-up surveys. The raw score of K10 ranges from 10 to 50 points, with higher scores indicating a higher level of psychological distress. We transformed the K10 score into z-scores based on the mean and SD.

We used the 10-item Social Support Rating Scale (SSRS) [38] to assess the level of an individual’s social support, which is the most prevalent tool for measuring social support in China [39]. The SSRS contains objective support (3 items about visible, practical, and direct support), subjective support (4 items about the perceived level of support from family members, neighbors, and friends), and social support (3 items about the level of social support used). The SSRS has been shown to have good reliability and validity [40] in China, with Cronbach α>.7 for both baseline and follow-up surveys in our study. The raw SSRS score ranges from 12 to 66 points, with higher scores indicating higher levels of social support. In this study, we transformed raw SSRS scores into z-scores. A CSS score was computed by aggregating the standardized measures (z-scores) of the individual-level social support within each community, which reflected the average levels of social support for each community. This method has been proven to be valid and widely applied in previous studies related to social support [30,41,42].

In this study, frailty was assessed using the criteria of the frailty phenotype, which was proposed and validated by Fried et al [43]. It consists of 5 components: shrinking (unintentional weight loss), slowness (a walking time of 4.6 m adjusted by gender and height; individuals who met the criteria in the walking test or were unable to perform the test due to physical limitations were considered positive for slowness) [44], self-reported exhaustion, weakness (grip strength), and low physical activity. Older people who met 3-5 criteria were considered frail, while those who met 0-2 criteria were considered nonfrail.

Multimorbidity was defined as the coexistence of 2 or more chronic health conditions based on the Chinese Centers for Disease Control and Prevention (CDC) recommendations [45] and previous studies [46,47], including hypertension, diabetes, chronic lung disease, heart disease, asthma, liver disease, stroke, dyslipidemia, cancer, digestive disease, kidney disease, and arthritis. To validate the accuracy of this information, trained interviewers asked for help from village doctors to confirm the self-reported chronic condition information in the chronic disease case management system. We categorized multimorbidity into 3 groups: no chronic condition, 1 chronic condition, and multimorbidity.

The control variables included sex (male, female), age, marital status (divorced/widowed, married), educational attainment (illiteracy, primary school, junior school, high school or above), economic status (household income per capita; quartile 1 was the poorest and quartile 4 the richest), sedentary behavior (hours/day), smoking status (never/past, current smoking), drinking status (never/past, current drinking), and individual-level social support. We selected these variables as potential confounders based on existing studies [48-50].

### Statistical Analysis

First, we compared the baseline characteristics between respondents and nonrespondents using cross-tabulation with *t* tests for continuous variables and chi-square tests for categorical variables (see Table 1). To examine the combined effects of frailty and multimorbidity, we created a *categorical indicator* with the following 6 groups: (1) nonfrail without a chronic condition, (2) nonfrail with 1 chronic condition, (3) nonfrail with multimorbidity, (4) frail without a chronic condition, (5) frail with 1 chronic condition, and (6) frail with multimorbidity. Multilevel linear mixed effects models were used to quantify the strength of the longitudinal association between frailty and multimorbidity combinations and psychological distress using 2 waves of data for each participant (see model 1 in Table 2), and then, cross-level interactions between CSS and combined frailty and multimorbidity (see model 2 in Table 2) were included. The selected 3-level linear mixed effects modeling strategy was appropriate for repeated measures to account for the hierarchical structure of the data set that included survey time nested within individuals and individuals nested within communities. A random intercept was included in each model. Both model 1 and model 2 were adjusted for sex, age, education, economic status, marital status, sedentary time, smoking status, drinking status, individual-level social support, and survey time (to capture any individual-level idiosyncratic disturbances over time).

**Table 1:** Attrition analysis of selected baseline characteristics.

Characteristics			Analytical sample (N=2785)		Dropouts (N=458)
**Sex, n (%);** *t* **(** *df* **)/** *χ* ^2^ **(** *df* **)=0.030 (1),** *P* **=.86**					
	Male	1015 (36.45)		165 (36.03)	
	Female	1770 (63.55)		293 (63.97)	
Age (years), mean (SD); *t* (*df*)/*χ*^2^ (*df*)=1.345 (1), *P*=.18			69.19 (6.16)		68.78 (6.34)
**Education, n (%)** **;** *t* **(** *df* **)/** *χ* ^2^ **(** *df* **)=0.271 (3),** *P* **=.97**					
	Illiteracy	1164 (41.80)		189 (41.27)	
	Primary school	1076 (38.64)		182 (39.74)	
	Junior school	407 (14.61)		66 (14.41)	
	High school or above	138 (4.96)		21 (4.59)	
**Economic status, n (%)** **;** *t* **(** *df* **)/** *χ* ^2^ **(** *df* **)=1.323 (3),** *P* **=.72**					
	Quartile 1	692 (24.85)		110 (24.02)	
	Quartile 2	675 (24.24)		120 (26.20)	
	Quartile 3	720 (25.85)		110 (24.02)	
	Quartile 4	698 (25.06)		118 (25.76)	
**Marital status, n (%)** **;** *t* **(** *df* **)/** *χ* ^2^ **(** *df* **)=3.451 (1),** *P* **=.06**					
	Divorced/widowed	695 (24.96)		133 (29.04)	
	Married	2090 (75.04)		325 (70.96)	
Sedentary time, mean (SD); *t* (*df*)/*χ*^2^ (*df*)=0.114 (1), *P*=.91 (hours/day)			4.36 (2.01)		4.34 (1.90)
**Smoking status, n (%)** **;** *t* **(** *df* **)/** *χ* ^2^ **(** *df* **)=0.009 (1),** *P* **=.93**					
	Never/past	2202 (79.07)		363 (79.26)	
	Current	583 (20.93)		95 (20.74)	
**Drinking status, n (%)** **;** *t* **(** *df* **)/** *χ* ^2^ **(** *df* **)=1.473 (1),** *P* **=.22**					
	Never/past	2161 (77.59)		367 (80.13)	
	Current	624 (22.41)		91 (19.87)	
**Combined frailty and multimorbidity** **, n (%)** **;** *t* **(** *df* **)/** *χ* ^2^ **(** *df* **)=7.935 (5),** *P* **=.16**					
	Nonfrail, no chronic condition	684 (24.56)		129 (28.17)	
	Nonfrail, 1 chronic condition	856 (30.74)		142 (31.00)	
	Nonfrail, multimorbidity	745 (26.75)		104 (22.71)	
	Frail, no chronic condition	71 (2.55)		13 (2.84)	
	Frail, 1 chronic condition	171 (6.14)		36 (7.86)	
	Frail, multimorbidity	258 (9.26)		34 (7.42)	
K10^a^, mean (SD); *t* (*df*)/*χ*^2^ (*df*)=0.640 (1), *P*=.52			16.63 (7.44)		16.39 (7.60)
Individual social support, mean (SD); *t* (*df*)/*χ*^2^ (*df*)=0.515 (1), *P*=.61			43.10 (6.27)		42.94 (6.45)
CSS^b^, mean (SD); *t* (*df*)/*χ*^2^ (*df*)=–1.190 (1), *P*=.23			43.05 (2.94)		43.23 (2.78)

^a^K10: 10-item Kessler Psychological Distress Scale.

^b^CSS: community-level social support.

**Table 2:** Longitudinal associations between combined frailty and multimorbidity, CSS^a^, and psychological distress before and during the COVID-19 pandemic^b^.

Effects			Model 1					Model 2		
			β (95% CI)		*P* value		β (95% CI)			*P* value
**Fixed effects: combined frailty and multimorbidity**										
	Nonfrail, no chronic condition	Reference		N/A^c^		Reference			N/A	
	Nonfrail, 1 chronic condition	0.13 (0.07 to 0.19)		<.001		0.13 (0.07 to 0.18)			<.001	
	Nonfrail, multimorbidity	0.25 (0.19 to 0.31)		<.001		0.24 (0.18 to 0.31)			<.001	
	Frail, no chronic condition	0.40 (0.27 to 0.53)		<.001		0.40 (0.26 to 0.53)			<.001	
	Frail, 1 chronic condition	0.48 (0.38 to 0.57)		<.001		0.46 (0.37 to 0.56)			<.001	
	Frail, multimorbidity	0.68 (0.60 to 0.77)		<.001		0.67 (0.59 to 0.75)			<.001	
**Fixed effects:** **CSS** **(z-scores)**			–0.06 (–0.11 to –0.02)		.008		–0.02 (–0.08 to 0.03)			.42
	Nonfrail, no chronic condition × CSS	N/A		N/A		Reference			N/A	
	Nonfrail, 1 chronic condition × CSS	N/A		N/A		–0.04 (–0.09 to 0.01)			.14	
	Nonfrail, multimorbidity × CSS	N/A		N/A		–0.03 (–0.08 to 0.03)			.33	
	Frail, no chronic condition × CSS	N/A		N/A		–0.03 (–0.15 to 0.09)			.67	
	Frail, 1 chronic condition × CSS	N/A		N/A		–0.11 (–0.19 to –0.02)			.011	
	Frail, multimorbidity × CSS	N/A		N/A		–0.16 (–0.23 to –0.09)			<.001	
Fixed effects: Baseline			Reference		N/A		Reference			N/A
Fixed effects: Follow-up			0.09 (0.02 to 0.15)		.008		0.08 (0.02 to 0.15)			.012
Fixed effects: Intercept			0.63 (0.19 to 1.07)		.005		0.64 (0.20 to 1.08)			.005
**Random effects**										
	Community level	0.02 (0.01 to 0.04)		<.001		0.03 (0.01 to 0.05)			<.001	
	Individual level	0.54 (0.50 to 0.58)		<.001		0.54 (0.51 to 0.58)			<.001	
	Residual	0.30 (0.29 to 0.32)		<.001		0.30 (0.28 to 0.31)			<.001	

^a^CSS: community-level social support.

^b^In total, 41 observations were excluded due to having missing data. All models were adjusted for sex, age, education, economic status, marital status, sedentary time, smoking status, and drinking status.

^c^N/A: not applicable.

Next, to minimize the possible reverse causality, 2-level mixed effects models with a lagged dependent variable (LDV) were conducted to account for the cluster of participants within communities, and we also controlled the levels of psychological distress at baseline (see Table 3). Model 1 in Table 3 included follow-up psychological distress as the dependent variable and the baseline categorical indicator and the change in CSS between the 2 waves as the independent variable to examine whether baseline frailty and multimorbidity combinations predicted psychological distress during the COVID-19 pandemic. Model 2 in Table 3 included model 3 variables plus a 2-way cross-level interaction term between baseline frailty and multimorbidity combinations and the increased CSS. Both model 3 and model 4 were adjusted for baseline confounding variables, including sex, education, economic status, marital status, sedentary time, smoking status, drinking status, individual-level social support, and K10 scores, to test whether the increased CSS from baseline to follow-up mitigated the adverse impact of combined frailty and multimorbidity on psychological distress.

**Table 3:** Baseline combined frailty and multimorbidity and changes in CSS^a^ for predicting psychological distress at follow-up^b^.

Effects		Model 1		Model 2		
		β (95% CI)	*P* value	β (95% CI)	*P* value	
**Fixed effects: combined frailty and multimorbidity**						
	Nonfrail, no chronic condition	Reference	N/A^c^	Reference	N/A	
	Nonfrail, 1 chronic condition	0.08 (0.01 to 0.15)	.033	0.08 (0.01 to 0.15)	.027	
	Nonfrail, multimorbidity	0.13 (0.06 to 0.21)	<.001	0.14 (0.06 to 0.21)	<.001	
	Frail, no chronic condition	–0.03 (–0.20 to 0.15)	.76	–0.02 (–0.20 to 0.16)	0.84	
	Frail, 1 chronic condition	0.26 (0.14 to 0.38)	<.001	0.26 (0.14 to 0.39)	<.001	
	Frail, multimorbidity	0.32 (0.22 to 0.43)	<.001	0.33 (0.22 to 0.44)	<.001	
**Fixed effects:** **∆** **CSS** **(z-scores)**		–0.07 (–0.12 to –0.02)	.003	–0.03 (–0.10 to 0.03)	.34	
	Nonfrail, no chronic condition × ∆CSS	N/A	N/A	Reference	N/A	
	Nonfrail, 1 chronic condition × ∆CSS	N/A	N/A	–0.06 (–0.13 to 0.01)	.08	
	Nonfrail, multimorbidity × ∆CSS	N/A	N/A	–0.02 (–0.09 to 0.05)	.62	
	Frail, no chronic condition × ∆CSS	N/A	N/A	–0.07 (–0.21 to 0.08)	.38	
	Frail, 1 chronic condition × ∆CSS	N/A	N/A	–0.05 (–0.16 to 0.05)	.32	
	Frail, multimorbidity × ∆CSS	N/A	N/A	–0.11 (–0.22 to –0.01)	.035	
Fixed effects: Intercept		0.71 (0.30 to 1.12)	<.001	0.72 (0.31 to 1.13)	<.001	
**Random effects**						
	Community level	0.02 (0.01 to 0.04)	<.001	0.02 (0.01 to 0.04)	<.001	
	Residual	0.47 (0.44 to 0.49)	<.001	0.47 (0.44 to 0.49)	<.001	

^a^CSS: community-level social support.

^b^In total, 41 observations were excluded due to having missing data. All models were adjusted for baseline covariates (sex, education, economic status, marital status, sedentary time, smoking status, drinking status, and the 10-item Kessler Psychological Distress Scale [K10] score), as well as concern about COVID-19 and the likelihood of contracting COVID-19 at follow-up.

^c^N/A: not applicable.

We ran additional analyses under the same multilevel framework to see how confounders adjusted the models. From model 0 (the unadjusted model) through model 2 (fully adjusted model), the variance partition coefficient for the community level decreased from 3% to 2% and from 6% to 4% for longitudinal association and predictive models, respectively, which implies that our confounders explained about one-third of the community-level variance in worsened psychological distress. Next, we added the separate variable of frailty status and multimorbidity in a full model to show the independent effects of frailty and multimorbidity as a comparison. All analyses were performed using Stata 14.2 (Stata Corp LP).

## Results

### Descriptive Analyses

The baseline survey included 3243 older people from 58 communities. Of the 3243 respondents at baseline, 458 (14.12%) individuals were lost to follow-up because of the following reasons: outmigration with children (n=121, 26.4%), dropping out (n=295, 64.4%), and deaths (n=42, 9.2%). This yielded an analytic sample of 2785 (follow-up rate=85.88%) who participated in the 2 surveys. All characteristics (sex, age, education, economic status, marital status, sedentary time, smoking and drinking status, chronic condition, frailty, and social support) were similar between respondents (n=2785, 85.88%) and nonrespondents (n=458, 14.12%) at the follow-up survey (*P*>.05). Of the 2785 respondents, 1770 (63.55%) were female, the mean age was 69.19 (SD 6.16) years, 1164 (41.80%) had no education, and 2062 (74.04%) were married. The characteristics of the selected sample and dropouts are provided in Table 1. Psychological distress score values increased from a K10 score of 16.60 (SD 7.46) at baseline to 18.23 (SD 8.00) at the follow-up survey. The combination of frailty and multimorbidity was observed in 258 (9.26%) respondents, while 684 (24.56%) respondents were nonfrail without a chronic condition. Both individual-level social support and CSS decreased during follow-up (see Table 1).

### Multilevel Mixed Effects Models

**Figure 1:**
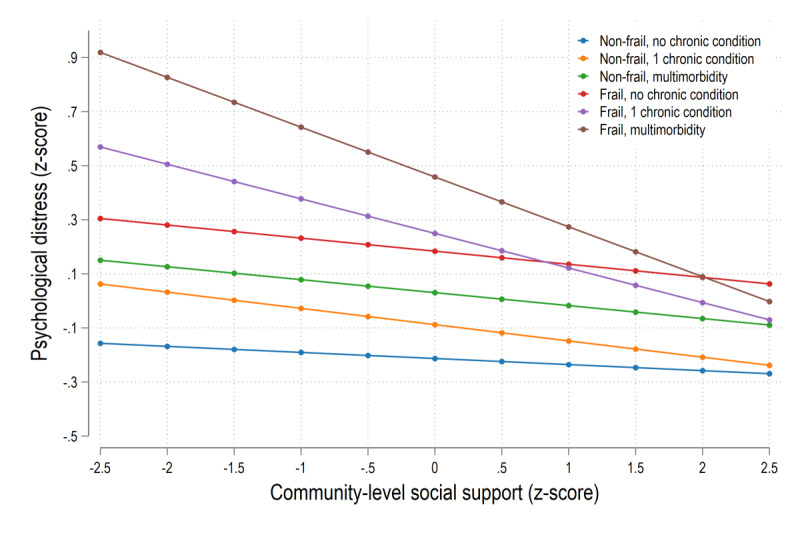
Longitudinal associations between CSS and psychological distress over 2 waves by combinations of frailty and multimorbidity (fully adjusted models). CSS: community-level social support.

Table 2 shows the results of multilevel mixed effects models with random intercepts at the individual and community levels. Model 1 showed that psychological distress significantly deteriorated during the COVID-19 pandemic compared with before the COVID-19 (β=.09, 95% CI 0.02-0.15, *P*=.008), while CSS (β=–.06, 95% CI –0.11 to –0.02, *P*=.008) was protectively associated with psychological distress. After adjustment for potential confounders, older adults who were nonfrail with 1 chronic condition (β=.13, 95% CI 0.07-0.19, *P*<.001), nonfrail with multimorbidity (β=.25, 95% CI 0.19-0.31, *P*<.001), frail without a chronic condition (β=.40, 95% CI 0.27-0.53, *P*<.001), and frail with 1 chronic condition (β=.48, 95% CI 0.38-0.57, *P*<.001) were at higher risk of psychological distress compared with older adults without any of the conditions, but the highest risk was observed in those with the combined presence of frailty and multimorbidity (β=.68, 95% CI 0.60-0.77, *P*<.001). As shown in model 2, the main effect of combined frailty and multimorbidity on psychological distress remained significant, and there was a significant cross-level interaction between CSS and frail individuals with multimorbidity (β=–.16, 95% CI –0.23 to –0.09, *P*<.001). To help the interpretations, the plots in Figure 1 show the estimated values of levels of psychological distress based on the estimates in model 2. Within the group of respondents who had combined frailty and multimorbidity, those with lower levels of CSS reported significantly higher psychological distress than respondents with higher levels of CSS, indicating the moderating role of CSS between frail individuals with multimorbidity and psychological distress.

**Figure 2:**
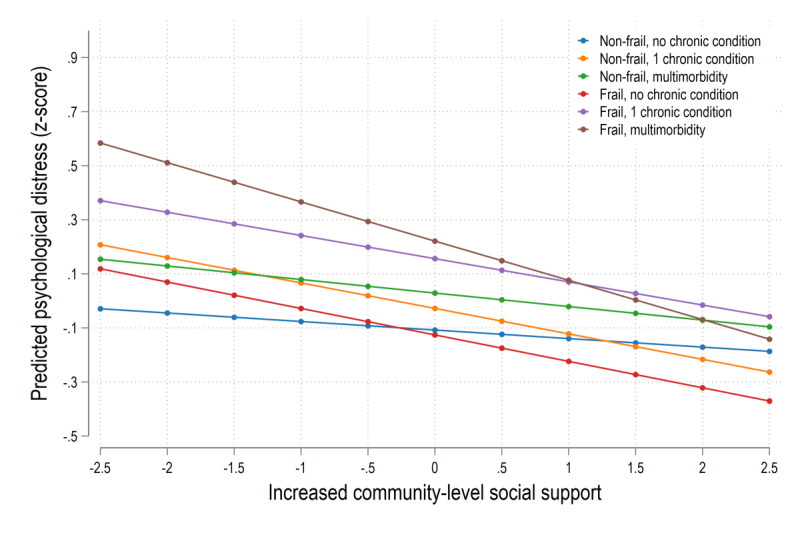
Increased CSS and psychological distress during the COVID-19 pandemic by baseline combinations of frailty and multimorbidity (fully adjusted models). CSS: community-level social support.

Model 1 in Table 3 shows the effects of baseline frailty and multimorbidity on wave 2 psychological distress. Those with coexisting frailty and multimorbidity (β=.32, 95% CI 0.22-0.43, *P*<.001) showed the strongest association with greater psychological distress during the COVID-19 pandemic compared with nonfrail individuals without a chronic condition, while increased CSS (β=–.07, 95% CI –0.12 to –0.02, *P*=.003) from wave 1 to wave 2 was significantly associated with lower subsequent levels of psychological distress. In model 2, we examined whether increased CSS moderated the adverse impact of baseline coexisting frailty and multimorbidity on psychological distress during the COVID-19 pandemic. We found a significant interaction between increased CSS from wave 1 to wave 2 and coexisting frailty and multimorbidity (β=–.11, 95% CI –0.22 to –0.01, *P*=.035) for wave 2 psychological distress. As Figure 2 demonstrates, for individuals with coexisting frailty and multimorbidity at baseline, those with higher increased levels of CSS reported significantly lower psychological distress than respondents with lower increased levels of CSS, while such effect was less substantial for older adults who were nonfrail without a chronic condition.

## Discussion

### Principal Findings

Using data from a cohort of community-dwelling older adults in rural China, this study identified that coexisting frailty and multimorbidity are significantly associated with greater levels of psychological distress and that baseline coexisting frailty and multimorbidity predicted higher levels of psychological distress during the COVID-19 pandemic. Further, residing in communities with a higher atmosphere of social support moderates the relationship of combined frailty and multimorbidity and psychological distress. We also emphasized the role of increased CSS in mitigating the adverse effects of combined frailty and multimorbidity on psychological distress during the COVID-19 pandemic. These results highlight the community social environment, more specifically CSS, as an important buffer that may mitigate the adverse impacts of combined frailty and multimorbidity on psychological distress.

Multimorbidity and frailty are 2 major adverse health conditions among older adults [14], and the 2 conditions are also risk factors for mental health [6,7,51]. However, there is no previous research that has explicitly examined their combined effects on psychological distress in older adults. Only 1 similar study has shown that the combination of frailty and multimorbidity is associated with physical limitation and mortality [52], and our study extends the literature for psychological health. In our sample, the results of longitudinal association showed that the magnitude of the coefficient of psychological distress was strongest for coexisting frailty and multimorbidity compared to only 1 or none of the conditions. This is where this study makes the first novel contribution to previous work: we identified a vulnerable group of older adults at increased risk of psychological distress. It is plausible that frail older adults already face a higher risk of poor resolution of homeostasis [53], plus the worry that results from multimorbidity, making them more vulnerable when coping with stressful situations and increasing the risk of psychological distress. Results from this prospective study also add evidence to the literature by identifying baseline combined frailty and multimorbidity as a predictor of greater psychological distress in older adults during the COVID-19 pandemic, emphasizing the importance of identifying and preventing coexisting frailty and multimorbidity in reducing psychological distress in older adults when facing major public health issues. We should interpret our findings incorporating the context of the pandemic. Unlike normal daily circumstances, the COVID-19 pandemic would expose people to more complicated and wide-ranging stressors. In addition to the primary stressor of the fear of infection, the pandemic has brought a plethora of secondary stressors, such as social isolation and resource scarcity [51]. These sources of stress could lead rural residents to face the potential disruption of basic medical services, especially the possibly reduced access to non–COVID-19 health care, which therefore adversely affects their psychological health. For example, older persons with frailty and multimorbidity had greater needs for basic medical services; however, the lockdown and polymerase chain reaction (PCR) testing policies led to worries about access to health care services, which in turn led to greater psychological distress. One evidence is that while the rural health care system did not break down during the quarantine, most of the rural residents chose to delay health care seeking due to worry about being infected [54]. Our findings suggested that older adults with frailty and multimorbidity needed more professional psychological support during the pandemic; however, the availability of psychological therapy or support was limited, especially in rural areas. Moreover, the current mass quarantines and restrictions to public transport have inevitably become a major barrier to accessing psychological therapy or support for older adults [55], which may also cause their increased psychological distress.

Importantly, our study identified that residing in a community with higher social support mitigates the relationship between coexisting frailty and multimorbidity and psychological distress. This finding adds to the literature documenting the stress-buffering role of CSS in psychological health. The moderating role of CSS remains significant even after controlling for individual-level social support, suggesting that there are contextual effects that alleviate psychological distress regardless of individual-level social support. In other words, older adults with lower individual-level social support could also benefit from their communities with a higher social support atmosphere. One possible explanation is social contagion [56]. CSS is meaningful in shaping community cohesion and connectedness [30], which is important for individuals to obtain the resources they need. Compared with living in communities with low levels of social support, health-related information may be disseminated faster in communities with higher levels of social support [57], which may increase the likelihood of people taking up healthy standards of behavior. Older adults with coexisting frailty and multimorbidity are more likely to collect and notice health-related information and resources that are provided by social contagion (health-related information, behavioral norms, etc) and thereby benefit more from social contagion, while those without any of the conditions may not pay much attention to the information and thus may not be sensitive to the community atmosphere. We noticed that the interaction between nonfrail older individuals with multimorbidity and CSS was not significant, while that between frail older individuals with multimorbidity and CSS was significant. This finding may be due to the different severity of disease within the multimorbidity group across the frailty state (ie, frail people may have more advanced diseases than nonfrail people). Older people with multimorbidity who have not yet developed a frail state may be resilient to the negative effect of multiple comorbidities. Thus, frail individuals with multimorbidity are more sensitive and pay more attention to their own health than nonfrail individuals with multimorbidity. Concerning the dynamic role of CSS, we observed that increased CSS from baseline to follow-up mitigated the adverse impact of baseline coexisting frailty and multimorbidity on psychological distress during the COVID-19 pandemic. Practically, our findings imply that interventions targeted toward improving CSS might alleviate the effects of frailty and multimorbidity on the psychological well-being of older adults, especially when facing major public health issues.

We also observed an increase in psychological distress in the context of the COVID-19 pandemic. In addition to the fear of being infected with COVID-19, widespread secondary stressors associated with the pandemic may include decreased opportunities for interpersonal contact and excursions and potential economic loss [51]. Given the lack of control groups that were unaffected by the COVID-19 pandemic in our study, more complex statistical design is needed in future studies, and we will not discuss more at this point.

### Strengths and Limitations

This study has several strengths and limitations. The novelties and strengths of this study include the study design and the measures we used. First, we used a large sample of community-dwelling older adults and followed up the psychological health outcomes with little missing data. Moreover, we used repeated measures of the key predictors and outcomes, including frailty, chronic conditions, social support, and psychological distress, and adjudicated K10 and CSS using standardized criteria. Furthermore, we not only used 2 waves of the data to show the longitudinal association between coexisting frailty and multimorbidity and psychological distress but also used baseline values to predicted psychological distress during the COVID-19 pandemic, which showed the robustness of the results.

Some limitations need to be considered. First, the observed relationships between combined frailty and multimorbidity and psychological distress cannot be constructed as causal relations, because this was an observational study. However, we took a set of measures to minimize the possible reverse causality, such as using lagged dependent variable models, controlling psychological distress at baseline, and adding a wave variable. Second, this study was conducted only in rural areas; whether the results are applicable to urban areas needs further study. In addition, our sample may not be representative of all rural older adults in China, as our participants were selected from 3 counties. As such, more nationally representative studies should be conducted to corroborate our findings. Third, as social support was assessed based on questionnaires, the results are subject to measurement bias: individuals with severe psychological distress might perceive themselves as having poor social support. However, we speculate that the aggregating social support at the community level could be influenced less. Fourth, some variables, such as chronic conditions and sedentary time, were self-reported, which may result in some recall bias. Finally, we cannot completely rule out residual confounding because of unmeasured variables; however, our results were robust after adjustment for multiple confounding variables.

### Conclusion

In summary, the findings of this study indicate that community-dwelling rural older adults with both frailty and multimorbidity have higher levels of psychological distress compared to those with either condition alone or none of the conditions. Baseline coexisting frailty and multimorbidity predict the most subsequent psychological distress during the COVID-19 pandemic. Thus, frail rural older adults with multimorbidity constitute a high-risk group for psychological distress. Moreover, residing in communities with highly average levels of social support moderates the relationship between combined frailty and multimorbidity and psychological distress, and we confirmed the role of increased CSS in buffering the adverse effects of combined frailty and multimorbidity on psychological distress during the COVID-19 pandemic. Our findings suggest that more public health and clinical attention should be paid to frail older adults with multimorbidity in response to psychological distress. In addition, improving the average levels of social support within communities may be an effective approach to alleviating psychological distress in older adults who concurrently manifest frailty and multimorbidity.

## Abbreviations

CSS: community-level social support
K10: 10-item Kessler Psychological Distress Scale
NICE: National Institute for Health and Care Excellence
SSRS: Social Support Rating Scale

## Data Availability

The data sets used in this study are not publicly available due to them containing information that could compromise research participant privacy, but they are available from the corresponding author upon reasonable request.
